# Supplementary material for: Highly efficient generation of isogenic pluripotent stem cell models using prime editing
Source: eLife. 2022 Sep 7;11:e79208. doi: 10.7554/eLife.79208 (PMC9584603; doi:10.7554/eLife.79208)
Supplement: Figure 2—figure supplement 2—source data 1. [file elife-79208-fig2-figsupp2-data1.zip › Figure 2 ΓÇöfigure supplement 2 source data.pdf]

# Figure 2 —figure supplement 2 source data

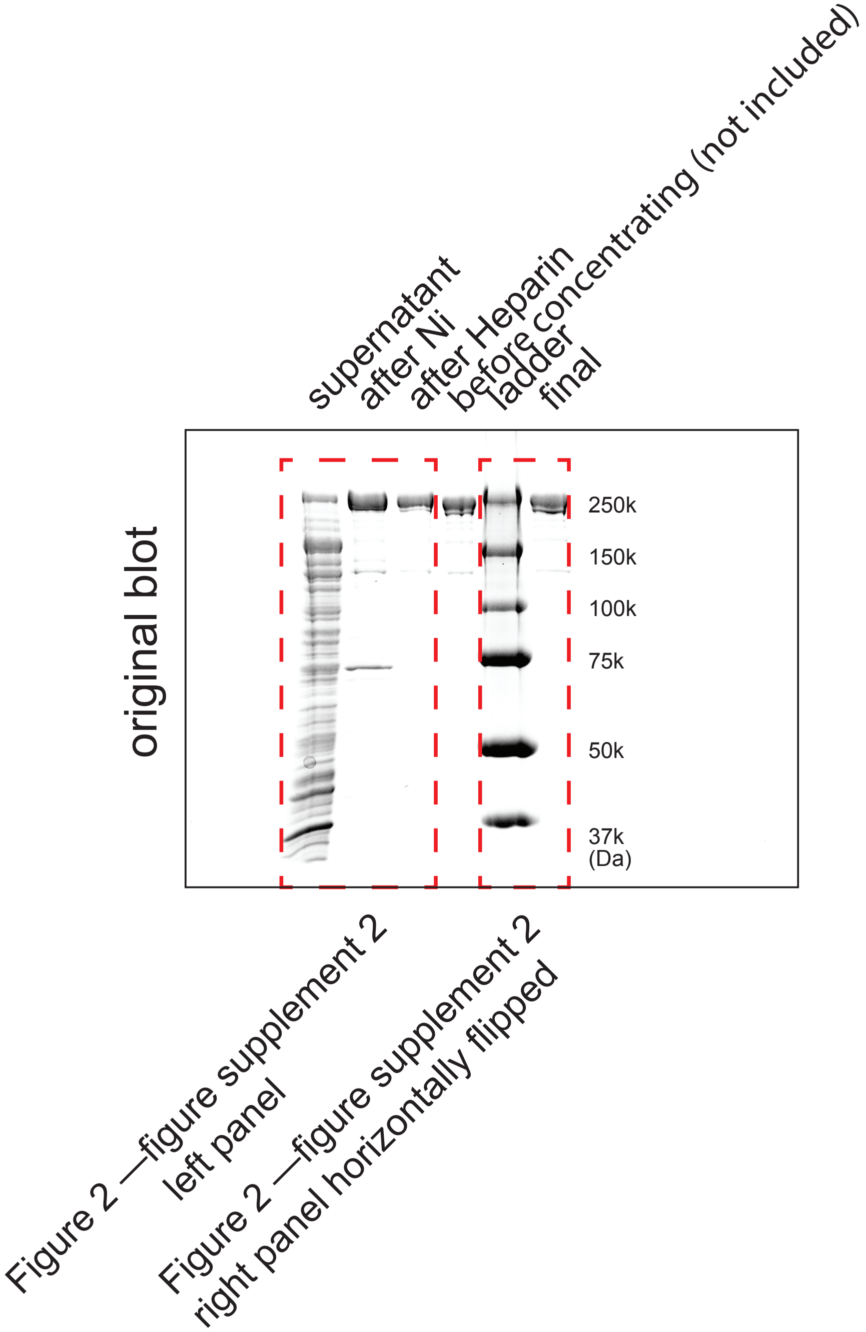


!"#$%&'()  *"#$%&'+$,,-&.&/0 '( !
