# Supplementary material for: Highly efficient generation of isogenic pluripotent stem cell models using prime editing
Source: eLife. 2022 Sep 7;11:e79208. doi: 10.7554/eLife.79208 (PMC9584603; doi:10.7554/eLife.79208)
Supplement: Supplementary file 1. [file elife-79208-supp1.docx]

**Supplementary File 1:** Table providing DNA Oligonucleotides and gBlocks gene fragment sequences**.**

| **Oligo Name** | **Purpose** | **Sequence** |
| --- | --- | --- |
| SP-HEK3 | NGS genotyping, HEK3 locus | GCTCTTCCGATCTCAGGTCCCTCCTCTCCTGG |
| ASP-HEK3 | NGS genotyping, HEK3 locus | GCTCTTCCGATCTTGGCCTGGGTCAATCCTTG |
| SP-LRRK2 | NGS genotyping, LRRK2 locus | GCTCTTCCGATCTTTTTGATGCTTGACATAGTGGAC |
| ASP-LRRK2 | NGS genotyping, LRRK2 locus | GCTCTTCCGATCTCACATCTGAGGTCAGTGGTTATC |
| SP-SNCA-A30P-1 | NGS genotyping, SNCA-A30P locus | GCTCTTCCGATCTTGTTTTCCAGTGTGGTGTAAAGG |
| ASP-SNCA-A30P-1 | NGS genotyping, SNCA-A30P locus | GCTCTTCCGATCTCCATCACTCATGAACAAGCACC |
| SP-SNCA-A30P-2 | NGS genotyping, SNCA-A30P | GCTCTTCCGATCTAAAGGCCAAGGAGGGAGTTG |
| ASP-SNCA-A30P-2 | NGS genotyping, SNCA-A30P locus | GCTCTTCCGATCTCAGTCCACCTTTTTGACAAGCA |
| SP-SNCA-A53T | NGS genotyping, SNCA-A53T locus | GCTCTTCCGATCTGTGGTGGTTACTGGAGTTCCTT |
| ASP-SNCA-A53T | NGS genotyping, SNCA-A53T locus | GCTCTTCCGATCTTCTTGAATACTGGGCCACAC |
| SP-LRRK2-RLFP | RLFP genotyping, LRRK2 locus | TTTTGATGCTTGACATAGTGGAC |
| ASP-LRRK2-RLFP | RLFP genotyping, LRRK2 locus | CACATCTGAGGTCAGTGGTTATC |
| SP-AAVS1-HR-L | Genotyping, *AAVS1* knock-in | CCCGCTTCAGTGACAACGTC |
| ASP-AAVS1-HR-L | Genotyping, *AAVS1* knock-in | GAACTCTGCCCTCTAACGCT |
| SP-AAVS1-HR-R | Genotyping, *AAVS1* knock-in | TGCATCGCATTGTCTGAGTAG |
| ASP-AAVS1-HR-R | Genotyping, *AAVS1* knock-in | TACCCCGAAGAGTGAGTTTGC |
| o-LRRK2-G2019S (CRISPR/CAS9) | HDR template for CRISPR/Cas9-baesd LRRK2 (G2019S) targeting | CCTGGTGTGCCCTCTGATGTTTTTATCCCCATTCTACAGCAGTACTGAGCAATGCTGTAGTCAGCAATCTTTGCAATGATGGCAGCATTGGGATACAGTGTGAAAAGCAG |
| o-LRRK2-G2019S (TALEN) | HDR template for TALEN-based LRRK2 (G2019S) targeting | CTGCTTTTCACACTGTATCCCAATGCTGCCATCATTGCAAAGATTGCTGACTACAGCATTGCTCAGTACTGCTGTAGAATGGGGATAAAAACATCAGAGGGCACACCAGG |
| o-AAVS1-1 | bridging oligo for cloning AAVS1-SA-neo-CAGGS-nCas9-RT-2A-GFP plasmid | CCATCAGAAGCTGGGAGCTCCACCGCGGTGGCTAGCTAGTGCGGCCGCTAATACGACTCA |
| o-AAVS1-2 | bridging oligo for cloning AAVS1-SA-neo-CAGGS-nCas9-RT-2A-GFP plasmid | CCGGTCATCATCACCATCACCATTGAGTTTAATTCCTCGAGTCTAGAGGGCCCGTTTAAA |
| gblock-pET30-1 | bridging gblock for cloning pET30a-nCas9-RT plasmid | GTGGTGGTGGTGGTGCTCGAGTGCGGCCGCGACTTTCCTCTTCTTCTTGGGCTCGAATTCGCTGCCGTCGGCGGTTCTTTTTGAGCCGCCAGAGGGTGATGAATTTTCTATGAGGAGGGTAGAGGTGTCTGGAGTCTCTGTGATGGCTGCCTTTCGGGCCGCTTGGTCAGCCATCCGGTTGCCTCTAGCCTCGGCGCTGTGTCCCTTTTGATGTCCTGGACAATGGATTATGCTAAGTCTTTTGGGCAGAAAGAGGGCTTTTAGTAGGGCCAAGATCTCGTCTTTATTTTTGATCTCTT |
| gblock-pET30-2 | bridging gblock for cloning pET30a-nCas9-RT plasmid | CCACCTTGGCCATCTCGTTGCTGAAGATCTCTTGCAGATAGCAGATCCGGTTCTTCCGTCTGGTGTATCTTCTTCTGGCGGTTCTCTTCAGCCGGGTGGCCTCGGCTGTTTCGCCGCTGTCGAACAGCAGGGCTCCGATCAGGTTCTTCTTGATGCTGTGCCGGTCGGTGTTGCCCAGCACCTTGAATTTCTTGCTGGGCACCTTGTACTCGTCGGTGATCACGGCCCAGCCCACAGAGTTGGTGCCGATGTCCAGGCCGATGCTGTACTTCTTGTCGACTTTCCGCTTCTTCTTTGGTGACTCGAACTCGCTTCCGTCGGCTGTCCGTTTCATATGTATATCTCCTTCTTAAAGTTAAAC |
