# Supplementary material for: Highly efficient generation of isogenic pluripotent stem cell models using prime editing
Source: eLife. 2022 Sep 7;11:e79208. doi: 10.7554/eLife.79208 (PMC9584603; doi:10.7554/eLife.79208)
Supplement: Supplementary file 2. [file elife-79208-supp2.docx]

**Supplementary File 2:** Table providing list of generated plasmids and synthetic gRNA/ngRNA/pegRNA sequences.

| **pegRNAs, plasmid** | **plasmid name** | **protospacer** | **extension** | **RRID: Addgene** |
| --- | --- | --- | --- | --- |
| peg-HEK3-CTT-ins | pU6-pegRNA-HEK3-CTTins-px330-scaffold | GGCCCAGACTGAGCACGTGA | TCTGCCATCAAAGCGTGCTCAGTCTG | RRID:  Addgene_180017 |
| peg-LRRK2-G2019S, peg1 | pU6-pegRNA-LRRK2-G2019S-3a | GATTGCAAAGATTGCTGACTA | GCAATGCTGTAGTCAGCAATC | RRID:  Addgene_180432 |
| peg-LRRK2-G2019S, peg2 | pU6-pegRNA-LRRK2-G2019S-3b | GATTGCAAAGATTGCTGACTA | GCAATGCTGTAGTCAGCAATCTT | RRID:  Addgene_180433 |
| peg-LRRK2-G2019S, peg3 | pU6-pegRNA-LRRK2-G2019S-3c | GATTGCAAAGATTGCTGACTA | GCAATGCTGTAGTCAGCAATCTTTG | RRID:  Addgene_180434 |
| peg-LRRK2-G2019S, peg4 | pU6-pegRNA-LRRK2-G2019S-3d | GATTGCAAAGATTGCTGACTA | GCAATGCTGTAGTCAGCAATCTTTGCA | RRID:  Addgene_180435 |
| peg-SNCA-A30P | pU6-pegRNA-SNCA-A30P | CAGGGTGTGGCAGAAGCAGC | GTCTTTCCTGGTGCTTCTGCCACACC | RRID:  Addgene_180016 |
| peg-SNCA-A53T | pU6-pegRNA-SNCA-A53T | GGAGGGAGTGGTGCATGGTG | TTACCTGTTGTCACACCATGCACCACTCCC | RRID:  Addgene_181738 |
|  |  |  |  |  |
| **ngRNAs, plasmid** | **plasmid name** | **protospacer** |  | **RRID: Addgene** |
| ng-HEK3-CTT-ins | pBPK1520-HEK3-CTTins-ng | GTCAACCAGTATCCCGGTGC |  | RRID:  Addgene_180019 |
| ng-LRRK2-G2019S | BPK1520-LRRK2-G2019S-ng | GACAGACCTGATCACCTACC |  | RRID:  Addgene_180436 |
| ng-SNCA-A30P | pBPK1520-SNCA-A30P-ng | ACAAGCACCAAACTGACATT |  | RRID:  Addgene_180018 |
| ng-SNCA-A53T | pBPK1520-SNCA-A53T-ng | TCATAGGAATCTTGAATACT |  | RRID:  Addgene_181739 |
|  |  |  |  |  |
| **gRNAs, plasmid** | **plasmid name** | **protospacer** |  |  |
| gRNA-LRRK2-G2019S | Px330-EGFP-LRRK2-CRISPR/Cas9 | GATTGCAAAGATTGCTGACTA |  | RRID:  Addgene_180437 |
|  |  |  |  |  |
| **Other plasmids** | **plasmid name** |  |  |  |
| AAVS1-SA-neo-CAGGS-nCas9-RT-2A-GFP | AAVS1-SA-neo-CAGGS-PE2-2A-GFP |  |  | RRID:  Addgene_180014 |
| pET30a(+)-nCas9-RT | pET30a(+)-PE2 |  |  | RRID:  Addgene_180015 |
|  |  |  |  |  |
| **ngRNA, synthetic (Synthego)** |  | **protospacer** |  |  |
| ng-HEK3-CTT-ins |  | GTCAACCAGTATCCCGGTGC |  |  |
| ng-LRRK2-G2019S |  | GACAGACCTGATCACCTACC |  |  |
| ng-SNCA-A30P |  | ACAAGCACCAAACTGACATT |  |  |
| ng-SNCA-A53T |  | TCATAGGAATCTTGAATACT |  |  |
|  |  |  |  |  |
| **gRNA, synthetic (Synthego)** |  | **protospacer** |  |  |
| gRNA-AAVS1 |  | ACCCCACAGTGGGGCCACTA |  |  |
| **pegRNAs, customized synthetic, (Synthego or IDT)** |  | **pegRNA sequence** |  |  |
| peg-HEK3-CTT-ins |  | mG*mG*mC*rCrCrArGrArCrUrGrArGrCrArCrGrUrGrArGrUrUrUrUrArGrArGrCrUrArGrArArArUrArGrCrArArGrUrUrArArArArUrArArGrGrCrUrArGrUrCrCrGrUrUrArUrCrArArCrUrUrGrArArArArArGrUrGrGrCrArCrCrGrArGrUrCrGrGrUrGrCrUrCrUrGrCrCrArUrCrArArArGrCrGrUrGrCrUrCrArGrUrCrUrGrU*mU*mU*mU |  |  |
| peg-LRRK2-G2019S |  | mA*mU*mU*rGrCrArArArGrArUrUrGrCrUrGrArCrUrArGrUrUrUrUrArGrArGrCrUrArGrArArArUrArGrCrArArGrUrUrArArArArUrArArGrGrCrUrArGrUrCrCrGrUrUrArUrCrArArCrUrUrGrArArArArArGrUrGrGrCrArCrCrGrArGrUrCrGrGrUrGrCrGrCrArArUrGrCrUrGrUrArGrUrCrArGrCrArArUrCrUrUrUrGrU*mU*mU*mU |  |  |
| peg-SNCA-A30P |  | mC*mA*mG*rGrGrUrGrUrGrGrCrArGrArArGrCrArGrCrGrUrUrUrUrArGrArGrCrUrArGrArArArUrArGrCrArArGrUrUrArArArArUrArArGrGrCrUrArGrUrCrCrGrUrUrArUrCrArArCrUrUrGrArArArArArGrUrGrGrCrArCrCrGrArGrUrCrGrGrUrGrCrGrUrCrUrUrUrCrCrUrGrGrUrGrCrUrUrCrUrGrCrCrArCrArCrCrU*mU*mU*mU |  |  |
| peg-SNCA-A30P-correction |  | mC*mA*mG*rGrGrUrGrUrGrGrCrArGrArArGrCrArCrCrGrUrUrUrUrArGrArGrCrUrArGrArArArUrArGrCrArArGrUrUrArArArArUrArArGrGrCrUrArGrUrCrCrGrUrUrArUrCrArArCrUrUrGrArArArArArGrUrGrGrCrArCrCrGrArGrUrCrGrGrUrGrCrGrUrCrUrUrUrCrCrUrGrCrUrGrCrUrUrCrUrGrCrCrArCrArCrCrU*mU*mU*mU |  |  |
